# Identification and Characterization of Biomarkers and Their Role in Opioid Addiction by Integrated Bioinformatics Analysis

**DOI:** 10.3389/fnins.2020.608349

**Published:** 2020-11-27

**Authors:** Xiuning Zhang, Hailei Yu, Rui Bai, Chunling Ma

**Affiliations:** ^1^Hebei Key Laboratory of Forensic Medicine, Collaborative Innovation Center of Forensic Medical Molecular Identification, College of Forensic Medicine, Hebei Medical University, Shijiazhuang, China; ^2^Research Unit of Digestive Tract Microecosystem Pharmacology and Toxicology, Chinese Academy of Medical Sciences, Shijiazhuang, China; ^3^Department of Anesthesiology, The Third Hospital of Hebei Medical University, Shijiazhuang, China

**Keywords:** opioid addiction, biomarker, nucleus accumbens, ADCY9, conditioned place preference

## Abstract

Although numerous studies have confirmed that the mechanisms of opiate addiction include genetic and epigenetic aspects, the results of such studies are inconsistent. Here, we downloaded gene expression profiling information, GSE87823, from the Gene Expression Omnibus database. Samples from males between ages 19 and 35 were selected for analysis of differentially expressed genes (DEGs). Kyoto Encyclopedia of Genes and Genomes (KEGG) pathway and Gene Ontology (GO) enrichment analyses were used to analyze the pathways associated with the DEGs. We further constructed protein-protein interaction (PPI) networks using the STRING database and used 10 different calculation methods to validate the hub genes. Finally, we utilized the Basic Local Alignment Search Tool (BLAST) to identify the DEG with the highest sequence similarity in mouse and detected the change in expression of the hub genes in this animal model using RT-qPCR. We identified three key genes, *ADCY9*, *PECAM1*, and *IL4*. *ADCY9* expression decreased in the nucleus accumbens of opioid-addicted mice compared with control mice, which was consistent with the change seen in humans. The importance and originality of this study are provided by two aspects. Firstly, we used a variety of calculation methods to obtain hub genes; secondly, we exploited homology analysis to solve the difficult challenge that addiction-related experiments cannot be carried out in patients or healthy individuals. In short, this study not only explores potential biomarkers and therapeutic targets of opioid addiction but also provides new ideas for subsequent research on opioid addiction.

## Introduction

Opioid abuse is currently a severe global epidemic problem for public health (Rudd et al., 2016; Roxburgh et al., 2017). In 2015, data from the WHO database revealed 450,000 deaths due to drug misuse globally. Among these deaths, 168,000 were due to opioid overdose. Indeed, from 2000 to 2015, the mortality rates related to opioid overdose increased by 500%. Opioid overdose-related deaths increased by 11% between 2014 and 2015 alone (Frauger et al., 2017; Schifano et al., 2019), posing massive public health costs. It is therefore urgent to explore the mechanism of opioid addiction.

The impact of opioid abuse on the human body is a continuous process. Drug addiction has been recognized as a chronic relapsing disease of the brain, as drug abuse-induced addiction has a significant impact on the central nervous system. Constant exposure of the human brain to opioids results in changes at the epigenetic, mRNA, neuropeptide, and protein levels (Kendler et al., 2003; Agrawal and Lynskey, 2008; Nelson et al., 2013). Furthermore, these factors can affect the next generation through maternal drug abuse during pregnancy (Desai et al., 2015). Although there is no specific gene that can be used as a biomarker for an opioid use disorder, most current studies suggest that opioid-induced gene changes play roles at many different levels, directly affecting reward effects or drug metabolic pathways or by affecting the body’s negative emotions. For example, OPRM1, an essential nucleus in opioid addiction, can trigger opioid addiction by participating in the orchestration of rewarding effects and the desire to avoid withdrawal symptoms (Hearing et al., 2018). The core and shell of the Nucleus accumbens (NAc), respectively, create complex neuroprotection loops by communicating with brain regions such as the prefrontal cortex, hippocampus, and thalamus. A large number of studies have proved that NAc is closely related to drug-induced reward, psychological craving, reinforcement, and other effects (Cooper et al., 2017; De Jong et al., 2019). Therefore, based on the morphine-related conditioned reward memory animal model, this study took the NAc brain region as the research object, focusing on the regulation mechanism of the changes in the expression level of related genes during the formation of morphine addiction.

There are many risk factors for genetic and epigenetic changes leading to the formation of opioid addiction, including gender and age (Gwira Baumblatt et al., 2014; Kolodny et al., 2015; Chartoff and Mchugh, 2016; Webster, 2017; Blanco and Volkow, 2019). Although addiction can start at any age, adolescents and young people in their developmental stages are more likely to try new things, which is one of the reasons why young people, especially men, account for the majority of drug addicts (Santiago Rivera et al., 2018). Few studies have focused on a specific age-group or a single-sex group. Therefore, we hope that re-screening addiction-related factors identified in relevant studies from the existing community according to age and gender stratification will reveal new insights. To identify transcriptome changes caused by opioid addiction in the young male population and to determine the mechanism of opioid addiction, we analyzed their transcriptomes.

Up until now, most studies at the animal level have been aimed at a specific age group or a single-sex animal group. Due to ethical issues and the limitation of population differences, it is challenging to study the mechanisms of drug-use disorders in humans. Whether the results of animal studies can be applied to humans is also a key question worth considering. If the target molecules identified in animal studies are also highly conserved in humans, then we will be more confident that the results obtained from animals can more likely be applied to human diseases.

Therefore, this study not only analyzed highly correlated transcriptome changes in the young male population but also established an opioid addiction model at the animal level through homology analysis to validate these changes, providing a new way of studying the mechanism of differentially expressed genes (DEGs) in opioid addiction. Strategies that target specific genetic and epigenetic factors and novel non-opioid medications hold promise as future therapeutic interventions of opioid abuse. We hope that successfully increasing treatment options in the clinical toolbox will help break the historical pattern of recurring opioid epidemics.

## Materials and Methods

### Microarray Data and Groups

The gene expression profiling information GSE87823 was collected from the Gene Expression Omnibus (GEO)^1^ database. Samples derived from 22 heroin addicts and five control subjects were examined in this array (Platform: GPL96). There were two additional conditions for enrollment: male; 15–35 years old.

### Data Retrieval and Preparation

The GEO2R tool^2^ was used to identify DEGs online. The GEOquery and Limma packages of the R language were used to control the high-latitude characteristics of the datasets according to the false positive rate control method proposed by Benjamini and Hochberg. For this process, raw datasets were filtered to meet the cut-off criteria of | FC| > 2 and *p* < 0.01. Altered genes were analyzed using the heat map tool utilizing the online platform OmicShare^3^.

### Processing of DEGs on the KEGG Pathway and GO Platforms

Functional analyses of specific genes are often performed using the Database for Annotation, Visualization, and Integrated Discovery (DAVID)^4^. The DAVID platform was therefore exploited to perform Gene Ontology (GO) analysis and KEGG pathway enrichment analyses; *p* < 0.05 represented statistical significance. These analyses revealed downregulated and upregulated genes.

### Design of a Protein-Protein Interaction Network (PPI) and Performance of Module Analyses for DEGs

Understanding the molecular and metabolic mechanisms of addiction development requires knowledge regarding the functional interactions among proteins involved in such processes. The Search Tool for the Retrieval of Interacting Genes (STRING)^5^ is an essential platform used to investigate the interaction among known and unknown (predicted) proteins of multiple organisms. In this study, DEGs were analyzed using this software and a PPI network was constructed to visualize the results. An interaction score of 0.4 was set as the threshold. Finally, the top 30 hub genes in the PPI network were identified using 10 different calculation methods in the Cytoscape software (Cytoscape_v3.7.1). Genes that overlapped in the calculations using all 10 algorithms were considered for downstream analysis.

### Sequence Similarity Analysis of Key Genes Among Different Species

Sequence similarity analysis was carried out with the Basic Local Alignment Search Tool (BLAST)^6^, searching the nucleotide collection (nr/nt) in the database using Megablast (optimized for highly similar sequences). Key genes with percentage identity (Per. Ident) > 85% and query cover = 100% were selected for detecting their expression changes in the NAc of opioid-addicted mice.

### Morphine-Induced Conditioned Place Preference

The unbiased conditioned place preference (CPP) paradigm was conducted as reported in a previous study (Golden et al., 2016). Each mouse was handled for 15 min by the investigator before the start of CPP. The experimental apparatus was made of different floor textures (rough or smooth surface) and two conditioning chambers (20 × 20 × 40 cm each) with different stripes (horizontal or vertical), which provided two distinct conditioning environments. On day 1 (Pre-test), mice moved freely and explored the entire equipment for 15 min. During this time, the duration of time the mice spent in each conditioning chamber was recorded. Subsequently, the mice were assigned into groups of approximately equal initial bias for the drug-paired chambers based on the time spent in each chamber. The conditioning test covered the period from 2 to 7 days. For this experiment, mice of the control group were given saline (i.h.) in both chambers while mice in the morphine group were given saline in one chamber and morphine (10 mg/kg, i.h.) in the other for 45 min. Each session was performed 6 h after the previous one and was performed by the same experimenter. We used subcutaneous administration due to morphine intraperitoneal administration of morphine reduced the bioavailability compared to subcutaneous administration (Handal et al., 2002). At the end of the test on day 8, the mice were allowed to move freely in the chamber, and the duration spent in each chamber was recorded. Preference scores (sec) were determined as the difference in time spent in the drug-paired chamber (Figure 6H).

### Measurement of *ADCY9* Expression Using RT-PCR

Harvested NAc tissues were treated with RNAiso Plus (TAKARA BIO INC) to isolate total RNA following the manufacturer’s instructions. ABI Prism 7500 sequence detection system software was used for data analysis. mRNA levels of the *ADCY9* gene were normalized to those of *GAPDH*. 5′–3′ nucleotide sequences of primers are CCCTGCCCACCGTCCCTTC (ADCY9 Reverse) and CGAGCCTAAGACCAGCACCAAG (ADCY9 Forward). The GAPDH forward primer sequence was AGCTGAACGGGAAGCTCACT, while the reverse primer sequence was CAACGTAGGTCCACCACTGACACGTTG.

### Data Analysis

All data are shown as means ± SEM. The *t*-test was used to compare qPCR results between morphine-treated mice and control mice for data with normally distributed data. *p* < 0.05 was considered to be significant. All statistical analyses were performed using SPSS.

## Results

### Identification of DEGs

**FIGURE 1:**
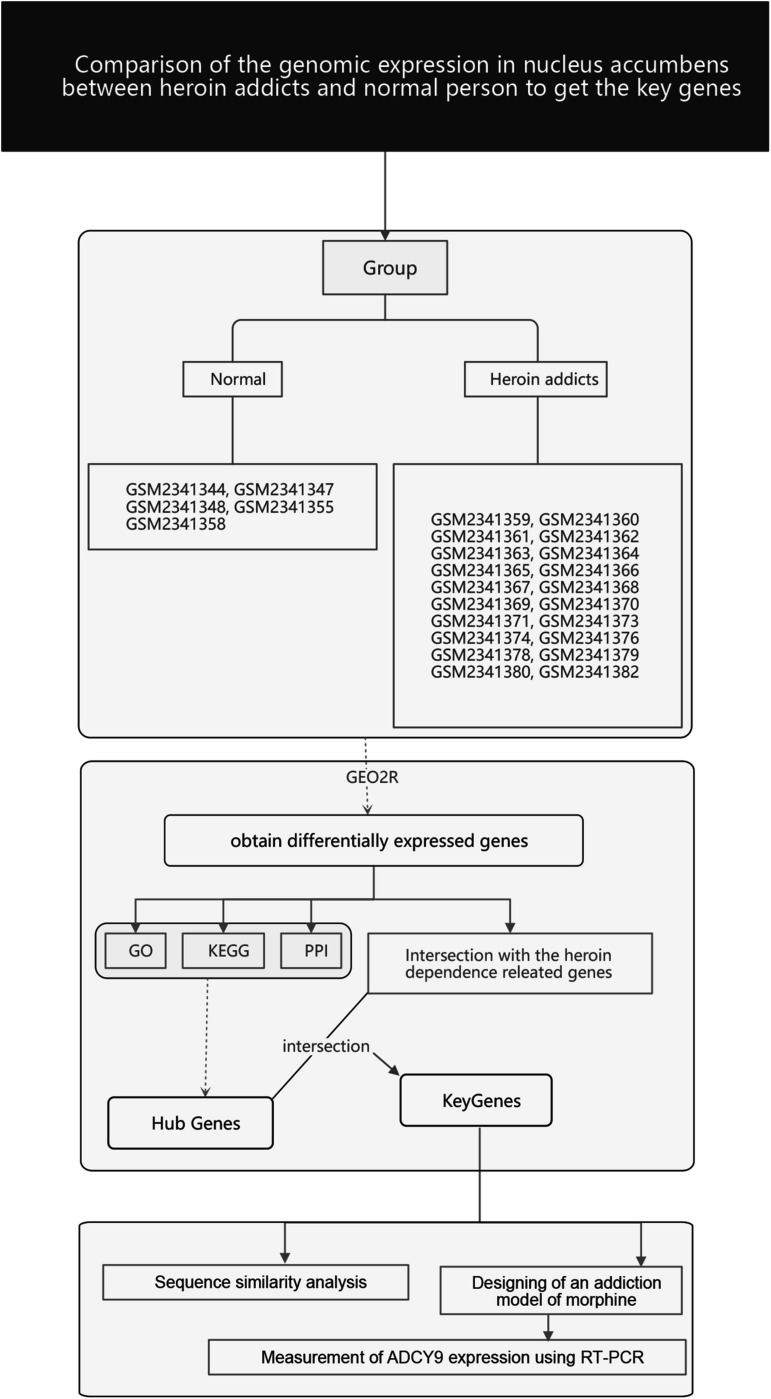
The flowchart of the bioinformatics analysis.

**FIGURE 2:**
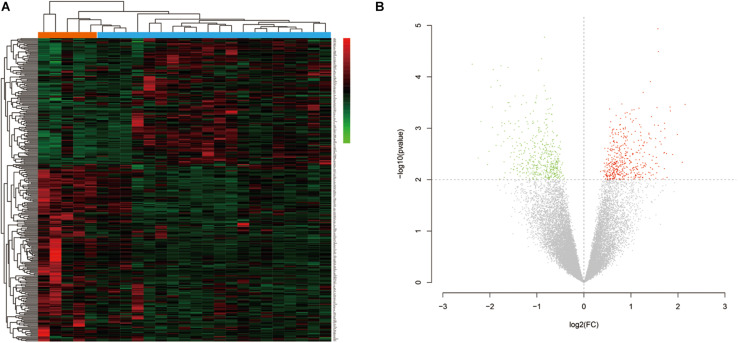
**(A)** Hierarchical-clustering heat map of the 269 differentially expressed genes. Red indicates upregulation, and green indicates downregulation. **(B)** Volcano plot of the identified differentially expressed genes (DEGs) distribution in GSE87823. Red indicates DEGS with log_2_FC ≥ 2, and green indicates DEGs with log_2_FC ≤ −2.

The research flow chart is illustrated in Figure 1. The age difference between the two groups was not statistically significant (Supplementary Table 6, *F* = 0.067, *p* = 0.799). We identified 289 DEGs in the nucleus accumbens (NAc) of heroin addicts compared with normal control NAc, including 166 downregulated and 123 upregulated DEGs (Figure 2).

### GO and KEGG Pathway Analysis

**FIGURE 3:**
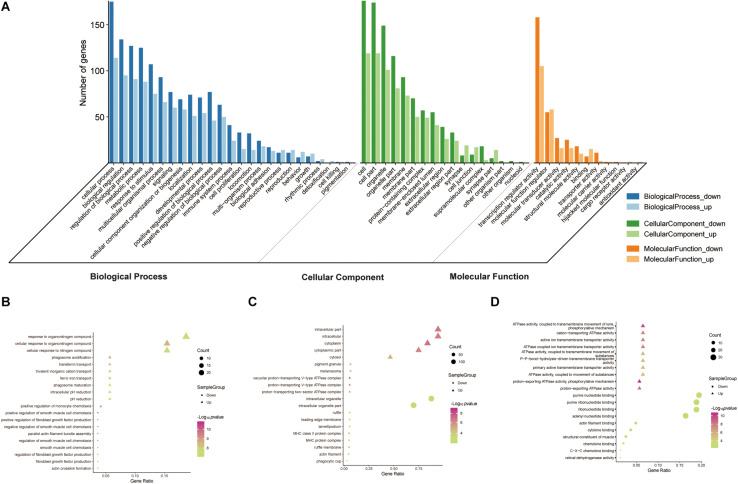
GO functional enrichment analysis of up- and downregulated DEGs. The genes were ordered according to their logFC values setting gene **(A)**. The top 10 terms of BP **(B)**, CC **(C)**, MF **(D)** by *p*-value. The gradual color represents the z-score.

**FIGURE 4:**
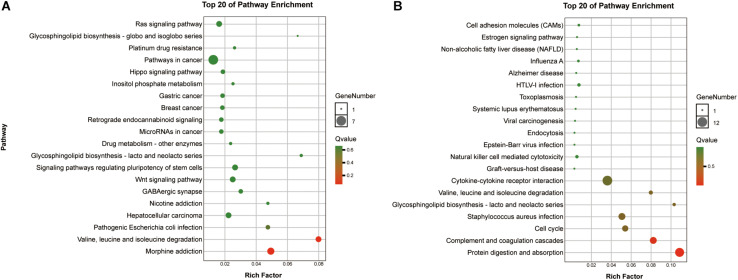
KEGG enrichment analysis of the top 20 up-DEGs **(A)** and down-DEGs **(B)** pathways: the gradual color represents the *p*-value, the size of the spots represents the gene numbers.

To reveal the roles of the DEGs, GO function enrichment was analyzed using the DAVID database. The top 10 cellular component, biological process, and molecular function (CC, BP, and MF) terms are shown in Supplementary Table S1 and Figure 3A. In the BP group, the upregulated DEGs were associated with phagosome acidification and maturation, cellular response to organonitrogen and nitrogen compounds, and transferrin transport. The downregulated DEGs were enriched in smooth muscle cell chemotaxis positive and negative regulation, fibroblast growth factor production, and regulation (Figure 3B). In the CC group, the upregulated DEGs were enriched in the terms vacuolar proton-transporting V-type ATPase complex, and cytoplasm, and the downregulated DEGs were enriched in the terms phagocytic cup and intracellular organelle (Figure 3C). Moreover, in the MF group, the upregulated DEGs were enriched in ATPase activity and the downregulated DEGs were enriched in actin filament and chemokine binding (Figure 3D). Pathway analysis revealed that upregulated DEGs were enriched in morphine addiction, nicotine addiction, endocannabinoid signaling, and GABAergic synapse pathways (Figure 4A) and downregulated DEGs were enriched in protein absorption and digestion, cell cycle, and cytokine-cytokine receptor interaction Alzheimer disease pathways (Figure 4B). Details are shown in Supplementary Table S2.

### PPI Network Analysis and Screening for Hub Genes

**FIGURE 5:**
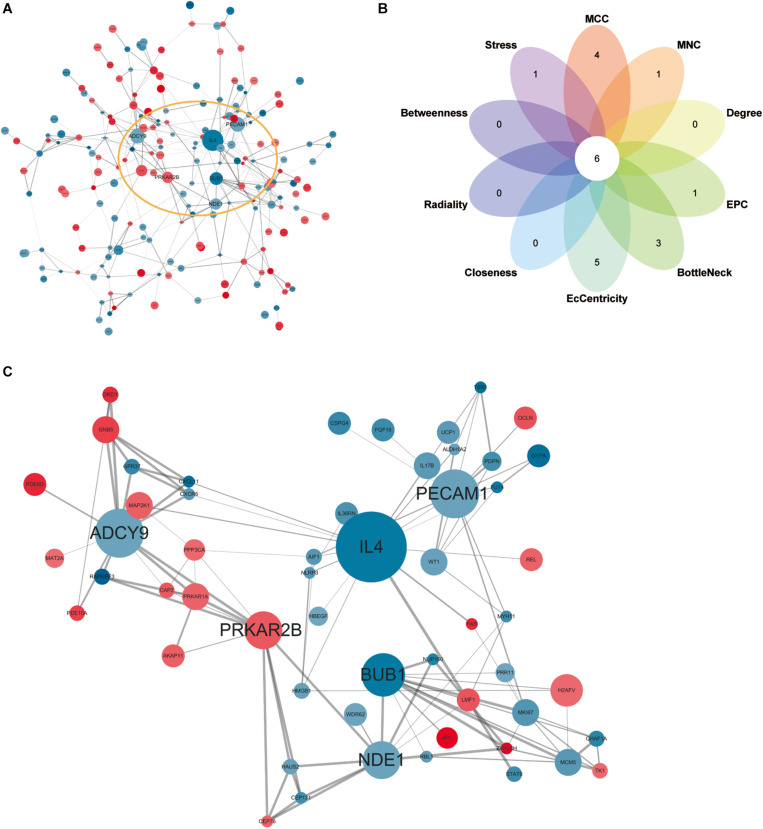
**(A)** Protein-protein interaction network for DEGs. Red, upregulated DEGs; blue, downregulated DEGs. The higher the degree value, the larger the node. **(B)**, the top 30 hub genes in the PPI network were identified by ten calculation methods and overlapped to obtain six key genes. **(C)** The PPI network of six key genes.

Network analysis using Cytoscape software and the STRING database yielded 156 nodes, among which 90 represented DEGs downregulated and 66 represented DEGs upregulated in heroin addicts compared with control individuals (Figure 5A). Using the plug-in CytoHubba in Cytoscape software, we determined scores based on 10 methods to screen for hub genes in the PPI network (Supplementary Table S3). We looked at the top 30 genes overlapping in each calculation and determined six key genes: *ADCY9*, *IL4*, *PECAM1*, *PRKAR2B*, *BUB1*, and *NDE1* (Figures 5B,C and Supplementary Table S4).

### Validation of Key Genes in NAc of Morphine-Addicted Mice

**FIGURE 6:**
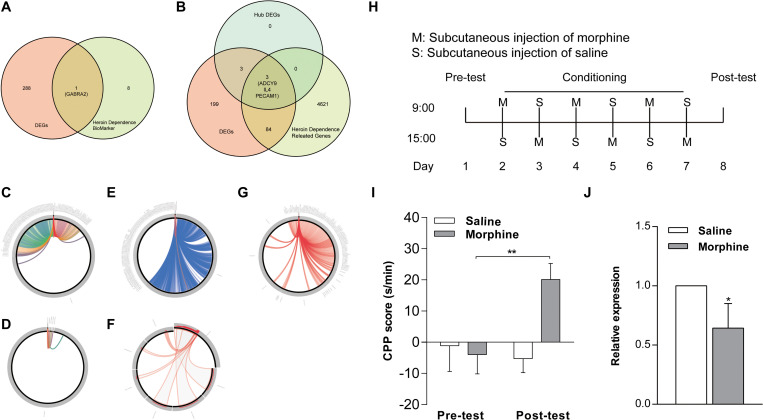
Validation of key genes in NAc of morphine-addicted mice. We found our DEGs by including one confirmed opioid addiction gene marker, GABRA2 **(A)**, and the results also included the genes ADCY9, IL4, and PECAM1, which we determined to be “key genes” **(B)**. We used BLAST to calculate the sequence similarity of 6 key genes between human and mouse **(D,F)** or human and other biological sequences **(C,E,G)**. **(H)** The timeline about the establishment of morphine addicted mice. **(I)** CPP induced by 10 mg/kg of morphine. The CPP score of **(J)**, mRNA levels of the ADCY9 gene in morphine addicted mouse’s NAc. *p < 0.05, **p < 0.01 significant difference.

We determined integrated gene-disease, chemical-disease, and chemical-gene interactions using the comparative toxicogenomics database^7^ to predict novel associations and create expanded networks (Davis et al., 2017). Using these data, we analyzed the relationships between DEGs and opioid addiction-related diseases. Our DEGs included one confirmed opioid addiction gene marker, *GABRA2* (Figure 6A), as well as the genes *ADCY9*, *IL4*, and *PECAM1*, which we determined to be “key genes” (Figure 6B). We used BLAST to calculate the sequence similarity of six key genes between human and mouse (Figures 6D,F) or human and other organisms (Figures 6C,E,G). The plots are shown in Figures 6C–F were created using Circoletto (Darzentas, 2010). Among these key genes, the human *ADCY9* sequence and the mouse *ADCY9* sequence had the highest similarity (Figure 6D).

To examine whether the expression of *ADCY9* would correlate with morphine addiction in mice, we conducted morphine-induced CPP, a wildly used morphine addiction model. Mice developed a significant place preference after morphine injection and these mice spent more time in the chamber where they were administered compared to mice that received saline after the conditioning phase. By contrast, the saline injection did not induce this effect (Figure 6I). Morphine also increased *ADCY9* mRNA expression in the mouse NAc, which was consistent with our previous results in human NAc (Figure 6J).

## Discussion

The pathogenesis of opioid abuse, a complex and chronic relapsing disease of the brain, remains unclear. Although numerous studies have confirmed that the mechanisms of opiate addiction include genetic and epigenetic aspects, the results of such studies are inconsistent (Skupio et al., 2017; Zhang et al., 2018). To our knowledge, our work is the first to use 10 different calculation methods, compared with the typical three methods, to calculate the association score among the DEGs for exploring novel hub genes. A recent study, GSE87823, detected the expression of genes in heroin abusers and healthy subjects. To minimize variability, we only enrolled samples from this dataset corresponding to males between the ages of 19 and 35 years, and genes exhibiting significant differential expression were identified using a variety of calculation methods. Our study provides evidence for the association of *ADCY9*, *PECAM1*, and *IL4* with heroin addiction through stringent bioinformatics analysis. The data presented here extend the previously reported association of *ADCY9*, *PECAM1*, and *IL4* with nicotine or alcohol addiction and psychiatric disorders. It is well known that the pharmacodynamically active metabolites of heroin include morphine, 6-diacetylmorphine, morphine 3-glucuronide, and morphine 6-glucuronide (Rook et al., 2006). In one study, five opioids were injected into heroin abusers, and morphine was found to make them feel as though they had received heroin (Comer et al., 2008). Moreover, there is some direct evidence that there are genes related to both heroin dependence and morphine dependence, such as *OPRM1* (Davis et al., 2019). Thus, we validated the transcriptional changes of *ADCY9* in a morphine addiction model in mice and found that these were consistent with those in humans. This study also provides evidence for the suitability of animal-level research in this regard, bypassing the difficulties in establishing human addiction models.

Our gene expression profiling identified pathways and key genes related to opioid addiction, which may potentially be therapeutic targets. Increased expression of *ADCY9* transcription is not only involved in the formation of a psycho-stimulant habit (Sokolov et al., 2003) but also found in the frontal pole brain of D2 mice after ethanol exposure (O’brien et al., 2018). A neuron is a special polarized cell type containing several synapses that respond to various stimuli (Wallace et al., 1998; Guzowski et al., 2001; Jones et al., 2001). The translation and transport of specific mRNA species regulate activity-dependent synaptic plasticity by modulating proteins that fine-tune neuronal responses to particular stimuli (Sutton and Schuman, 2006; Hoogenraad et al., 2007; Huang et al., 2017). We speculate that chronic opioid exposure causes changes in the synaptic plasticity of neurons by changing the transcription level of *ADCY9* in the NAc, thereby causing behavioral sensitization, which affects addictive behaviors. Opioid exposure also results in prolonged activation of N-methyl-D-aspartate (NMDA) receptors. NMDA inhibitor is linked to enhanced neuronal apoptosis in the developing rodent brain, and ADCY9 is involved in neuronal apoptosis induced by NMDA receptor blockade in neonatal rats (Sutton and Schuman, 2006; Hoogenraad et al., 2007; Huang et al., 2017). Of particular interest, immunoblotting data reveals a marked increase in GluN1 and GluN2B expression in three regions (medial prefrontal cortex mPFC, Lateral prefrontal cortex LPFC, and orbitofrontal cortex OFC) in men suffering from opioid addiction (Daneshparvar et al., 2019), suggesting that ADCY9 may also be involved in changes to NMDA receptors in opioid addiction. These changes may lead to behavioral sensitization and the formation of addictive memory. Due to ethical limitations, we cannot establish an opioid addiction model in humans to study the mechanism of ADCY9. However, we found that the similarity of the *ADCY9* gene sequence between mice and humans was more than 80%. By establishing a morphine addiction model in mice and detecting the expression level of *ADCY9* in the NAc, we found that the expression of *ADCY9* decreased. The expression of *ADCY9* in human opioid addiction showed the same trend. Therefore, we have reason to believe that it is beneficial to study the mechanism of ADCY9 in an opioid addiction mouse model.

IL4, an inflammatory factor, and PECAM1, platelet/endothelial cell adhesion molecule 1, both participate in the inflammatory reaction process of the body. Chronic morphine treatment induces an increase of IL4 in spleen cells (Greeneltch et al., 2005), and serum IL4 levels are also elevated in heroin and cocaine addicts (Ríos-Olivares et al., 2006). We know that the damage caused by opioid addiction is mostly related to the immune response, and previous studies have confirmed that an increase of PECAM1 in the blood-brain barrier of cocaine-addicted rats causes an immune response in endothelial cells, immune cells, and neuroendocrine cells, thus impairing the function of the blood-brain barrier (Fiala et al., 1998). In mice, IL4 and PECAM1 also cause neuroinflammation by recruiting mast cells and upregulating the release of various mediators, which may be involved in the formation of Parkinson’s disease (Hong et al., 2018). In addition, changes in serum PECAM1 levels contribute to the occurrence and development of autism and depression (Serebruany et al., 2005; Tsuchiya et al., 2007). IL4 and PECAM1 might serve as the molecular and cellular basis of neurological damage by opioid addiction.

In summary, investigating the specific genes regulating the development of opioid addiction is an essential component of early treatment of the disease. We provide evidence that ADCY9 may lead to behavioral sensitization and addictive memory formation by altering the synaptic plasticity of NAc neurons, while IL4 and PEAM1 may participate in neuroinflammation caused by opioid addiction through immune responses. To apply our research results to clinical treatment, we need further research to verify this mechanism. We will focus on validating the usefulness of these DEGs as diagnostic and/or prognostic markers in a subsequent study. Our research expands our understanding of the mechanism of opioid addiction and proposes possible targets for addressing the symptoms of opioid addiction. These results lay the foundation for further development of treatments and related research.

## Data Availability Statement

The datasets presented in this study can be found in online repositories. The names of the repository/repositories and accession number(s) can be found below: https://www.ncbi.nlm.nih.gov/geo/, GSE87823.

## Ethics Statement

The animal study was reviewed and approved by the Animal Care and Use Committee of Hebei medical University.

## Author Contributions

XZ conceived, designed, performed the experiments, analyzed the data, and wrote the manuscript. HY and RB performed the experiments and analyzed the data. CM conceived, designed and revised the manuscript. All authors contributed to the article and approved the submitted version.

## Conflict of Interest

The authors declare that the research was conducted in the absence of any commercial or financial relationships that could be construed as a potential conflict of interest.

## Footnotes

1 https://www.ncbi.nlm.nih.gov/geo/

2 https://www.ncbi.nlm.nih.gov/geo/geo2r/

3 http://www.omicshare.com/tools

4 https://david.ncifcrf.gov/

5 https://string-db.org/

6 https://blast.ncbi.nlm.nih.gov/Blast.cgi

7 http://ctdbase.org/

## Supplementary Material

Supplementary Table 1 GO terms enrichment for DEGs.

Supplementary Table 2 KEGG pathway analysis data of DEGs.

Supplementary Tables 3 and 4 10 methods to screen for hub genes.

Supplementary Table 5 Results of the 269 differentially expressed genes, and marked the key genes in the file with red font.

Supplementary Table 6 The demographic information of the normal people and addicts.
